# Older Korean men with inadequate vitamin D status have lower odds of radiologic osteoarthritis

**DOI:** 10.1038/s41598-022-15025-9

**Published:** 2022-07-05

**Authors:** Seunghee Kim, Gun-Woo Lee, Clara Y. Park

**Affiliations:** 1 Department of Food and Nutrition, Chonnam National University, 77 Yongbong-ro, Bukgu, Gwangju, 61186 South Korea; 2 Department of Orthopedic Surgery, Chonnam National University Medical School and Hospital, Gwangju, South Korea

**Keywords:** Musculoskeletal system, Rheumatic diseases, Endocrine system and metabolic diseases, Nutrition, Epidemiology

## Abstract

Most studies on osteoarthritis (OA) and vitamin D status were performed in Whites with relatively adequate vitamin D status. Associations may differ by baseline 25-hydroxyvitamin D (25(OH)D) and race. We assessed the odds of OA and joint pain according to vitamin D status in Korean adults ≥ 50 years of age in the nationally representative Korea National Health and Nutrition Examination Survey (*n* = 8575). Agreement between radiologic OA (ROA) and self-reported OA were also assessed. Multivariate logistic regression was performed and participants were stratified by sex. Adults with serum 25(OH)D < 12 ng/mL and 12 to < 20 ng/mL had 26% and 18% lower odds of knee ROA, respectively, compared to those with 25(OH)D ≥ 20 ng/mL. Similar results were observed in men, but not women. No associations were found between 25(OH)D and knee ROA severity, lumbar spine ROA, symptomatic OA, or knee pain. Sensitivity of self-reported OA was low (27%), indicating a weak possibility of reverse causation. Prospective studies are required to identify the possible causality of vitamin D on OA in Korean men.

## Introduction

Osteoarthritis (OA) is the most common joint disease affecting 240 million worldwide and a major cause of global years lived with disability^1^. Evidence that OA is a metabolic disorder, rather than local inflammation at the joint, is emerging^2^. Whether vitamin D can prevent or alleviate OA has been investigated but primarily focused on Whites with relatively high vitamin D status^3–9^. However, the pathology of OA may differ by race^10,11^. Genetic differences in OA and vitamin D metabolism have been reported to be associated with race^12,13^ while racial differences exist in pain sensitivity^14^. In addition, the null results of previous randomized controlled trials (RCTs) in Whites may be due to the relatively high mean baseline serum 25-hydroxyvitamin D (25(OH)D) levels of participants (≥ 20 ng/mL)^7–9^. A few studies point to a possible beneficial effect of vitamin D supplementation in OA patients with lower vitamin D status, but the sample sizes are inadequate for statistical analysis^9,15,16^. Therefore, the relationship between vitamin D and OA in Asians and in those with low vitamin D status may differ from Whites but studies with ample sample size are needed.

In Korea, the prevalence of OA is increasing with the rapid increase in the number of older adults^17^. In addition, the prevalence of low vitamin D status (25(OH)D < 20 ng/mL) is higher in Koreans than in Whites, reaching 48.1% and 65.9% in men and women ≥ 50 years of age, respectively^18^. The high prevalence of low vitamin D status in Koreans identify them as a suitable population to examine the relationship between low vitamin D status and OA. Therefore, we assessed the relationship between vitamin D status and OA prevalence, progression, and clinical symptoms in older Korean men and women using cross-sectional data from a nationally representative database. As this study is cross-sectional, we additionally assessed the agreement between ROA and self-reported OA to examine the possibility of behavioral effects on the association between vitamin D status and ROA due to knowledge of OA.

## Results

The distributions of general characteristics differed by ROA status for all covariates except for physical activity and season of blood draw (Table 1). Approximately 40.0% of men and 54.5% of women had ROA. Subjects with ROA tended to be older; have a higher BMI, lower income, and lower education level; be manual workers; drink less frequently; and have a higher vitamin D status than those without ROA. When stratified by sex, smoking status did not differ by ROA status within either sex.

**Table 1:** Characteristics of adults 50 years and older in KNHANES 2010–2013 by ROA status.

ROA	Total (*n* = 8575)			Men (*n* = 3830)			Women (*n* = 4745)		
	No	Yes	*P* value	No	Yes	*P* value	No	Yes	*P* value
n (%)	4128 (52.3)	4447 (47.7)		2138 (60.0)	1692 (40.0)		1990 (45.5)	2755 (54.5)	
Age (yr)	58.1 ± 0.2	64.5 ± 0.2	< 0.0001	58.7 ± 0.2	63.6 ± 0.3	< 0.0001	57.3 ± 0.2	65.1 ± 0.2	< 0.0001
BMI (kg/m^2^)	23.7 ± 0.1	24.6 ± 0.1	< 0.0001	23.8 ± 0.1	24.1 ± 0.1	0.01	23.6 ± 0.1	24.8 ± 0.1	< 0.0001
HEMI (%)	137.1 ± 4.9	98.4 ± 3.2	< 0.0001	143.1 ± 6.5	109.7 ± 5.5	< 0.0001	129.9 ± 4.5	90.9 ± 3.2	< 0.0001
**Education**									
≤ Elementary graduate	1320 (31.4)	2558 (54.0)	< 0.0001	532 (24.3)	647 (35.2)	< 0.0001	788 (39.9)	1911 (66.4)	< 0.0001
Middle school graduate	788 (20.7)	708 (16.9)		397 (19.3)	326 (21.1)		391 (22.3)	382 (14.2)	
≥ High school graduate	2020 (47.9)	1181 (29.1)		1209 (56.4)	719 (43.7)		811 (37.8)	462 (19.5)	
**Longest job**									
Manual work	1933 (48.7)	2664 (59.9)	< 0.0001	1131 (55.2)	1099 (65.7)	< 0.0001	860 (41.2)	1663 (56.0)	< 0.0001
Clerical work	2036 (47.7)	1514 (35.1)		1031 (44.8)	624 (34.3)		1051 (51.2)	944 (36.0)	
Other^a^	159 (3.6)	269 (5.0)					164 (7.6)	282 (8.1)	
Physical activity (min/wk)	631.2 ± 21.2	586.7 ± 27.7	0.17	733.3 ± 30.8	734.2 ± 42.6	0.99	509.9 ± 23.2	488.9 ± 25.6	0.51
**Smoking status**									
Current/previous smoker	1957 (50.1)	1614 (38.4)	< 0.0001	1838 (85.5)	1419 (85.1)	0.80	119 (7.9)	195 (7.4)	0.65
Never smoker	2171 (49.9)	2833 (61.6)		300 (14.5)	273 (14.9)		1871 (92.1)	2560 (92.6)	
**Alcohol consumption**									
≥ Once/month	2091 (53.8)	1759 (42.4)	< 0.0001	1511 (73.1)	1118 (68.4)	0.03	580 (30.8)	641 (25.1)	0.004
< Once/month	2037 (46.2)	2688 (57.6)		627 (26.9)	574 (31.6)		1410 (69.2)	2114 (74.9)	
**Menopausal status**									
Post-menopause							1759 (86.3)	2671 (96.2)	< 0.0001
Pre-menopause							231 (13.7)	84 (3.8)	
**Season of blood draw**									
May–December	2007 (50.4)	2122 (47.3)	0.076	1043 (51.1)	817 (47.8)	0.17	964 (49.7)	1305 (46.9)	0.26
June–November	2121 (49.6)	2325 (52.7)		1095 (48.9)	875 (52.2)		1026 (50.3)	1450 (53.1)	
**25(OH)D (ng/mL)**^**b**^	18.5 ± 0.2	19.3 ± 0.2	0.002	19.1 ± 0.2	20.5 ± 0.3	0.0002	17.7 ± 0.3	18.5 ± 0.2	0.03
< 12	563 (14.3)	580 (14.0)	0.001	220 (11.5)	139 (10.2)	0.002	343 (17.7)	441 (16.5)	0.01
≥ 12, < 20	2094 (50.0)	2085 (44.7)		1032 (48.0)	734 (41.0)		1062 (52.4)	1351 (47.2)	
≥ 20	1471 (35.7)	1782 (41.3)		886 (40.5)	819 (48.8)		585 (29.9)	963 (36.3)	

Joints assessed for ROA include the knee, lumbar spine, and hip. Data are shown as weighted mean ± standard error or unweighted *n* (weighted percentage). Differences in characteristics were investigated with weighted Rao-Scott χ^2^ or independent *t*-test. The total percentages may exceed 100% due to rounding. Sample weights were applied according to the directions of the KNHANES. BMI, body mass index; HEMI, household equivalent median income; KNHANES, Korea National Health and Nutrition Examination Survey; ROA, radiologic osteoarthritis; wk, week; yr, year; 25(OH)D, 25-hydroxyvitamin D.

^a^Includes unemployed and housewives.

^b^Serum 25(OH)D was log-transformed due to the absence of normality.

**Figure 1:**
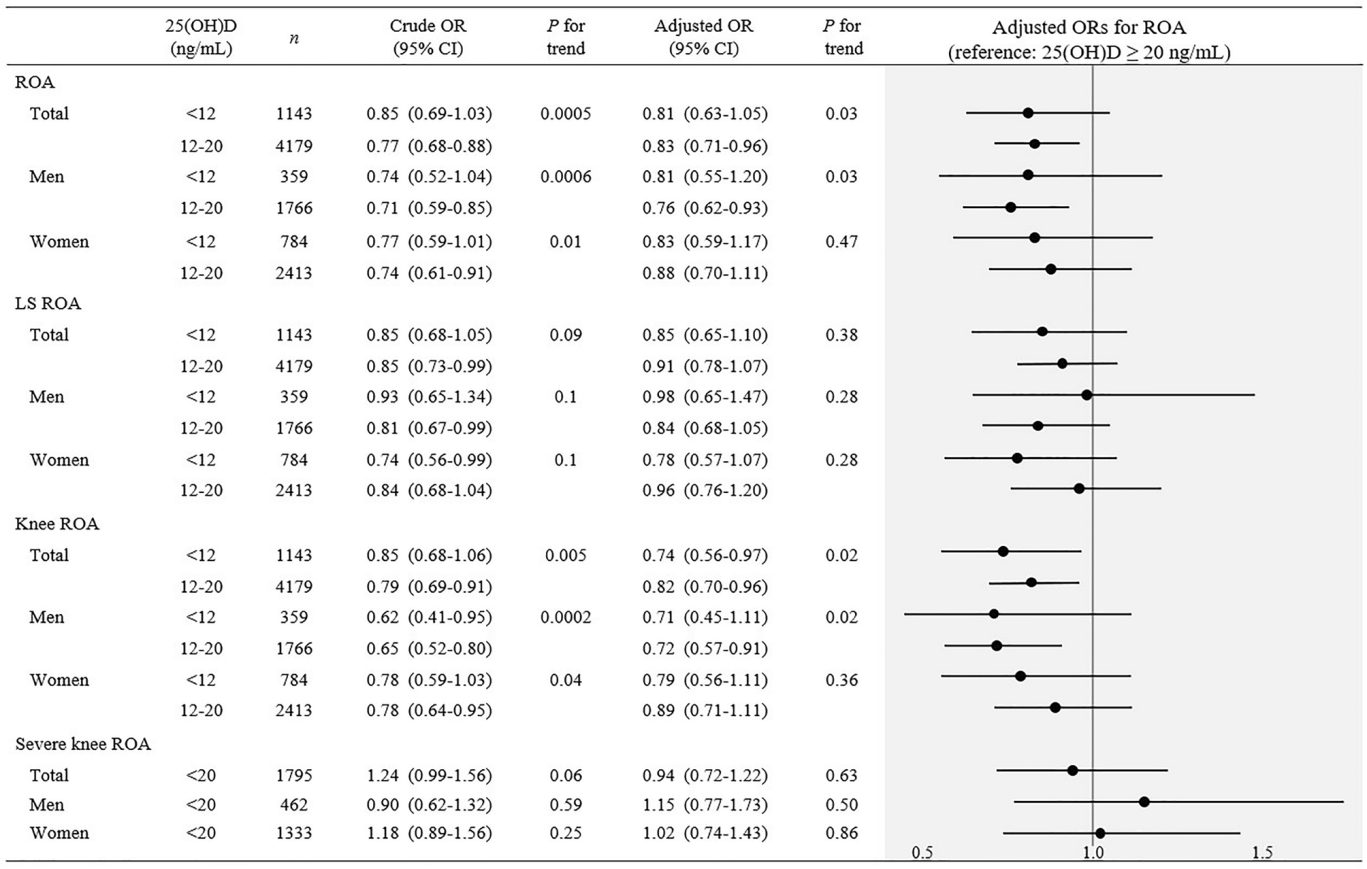
Crude and adjusted ORs (95% CI) of ROA by 25(OH)D status in adults 50 years and older from KNHANES 2010–2013. ROA indicates at least one incidence of ROA at the knee, lumbar spine, or hip confirmed by X-ray. Risk of severe knee ROA was assessed as the risk of KL grade ≥ 3 compared to KL grade 2. Sample size for the reference groups (25(OH)D ≥ 20 ng/mL) of ROA, LS ROA, and knee ROA for total, men, and women was 3253, 1705, and 1548, respectively. Vitamin D status to assess knee ROA severity was categorized into 2 groups due to the small sample size. The number of total, men, and women with 25(OH)D ≥ 20 ng/mL for knee ROA severity analysis was 1190, 476, and 714, respectively. Logistic regression was used for analyses. All models were adjusted for clustering and stratification. Adjusted models were adjusted for sex (total population), age, income, education level, longest job, physical activity, smoking status, alcohol consumption, body mass index, season of blood draw, survey year, and menopausal status (women). Weights were applied according to the guidelines of the KNHANES. CI, confidence interval; KL, Kellgren–Lawrence; KNHANES, Korea National Health and Nutrition Examination Survey; LS, lumbar spine; OR, odds ratio; ROA, radiologic osteoarthritis; 25(OH)D, 25-hydroxyvitamin D.

Adults with 25(OH)D < 20 ng/mL had lower odds of ROA (OR: 0.81 [95% CI: 0.63–1.05] and 0.83 [95% CI 0.71–0.96] for < 12 and 12 to < 20 ng/mL, respectively; *P* for trend = 0.03) compared to those with 25(OH)D ≥ 20 ng/mL (Fig. 1). When stratified by sex, this association remained in men only (*P* for trend = 0.03). Regarding knee ROA, adults with serum 25(OH)D < 12 and 12 to < 20 ng/mL had 26% (95% CI 0.56–0.97) and 18% (95% CI 0.70–0.96) lower risk of knee ROA, respectively (*P* for trend = 0.02), than participants with higher vitamin D status. Analyses by sex resulted in similar associations in men but not women. When additionally stratified by age or BMI, these results persisted in men younger than 65 years of age and in overweight/obese men (BMI ≥ 23) but not in men 65 years and older or underweight/normal weight men (BMI < 23; Table 2). Few associations were observed in women (Supplementary Table 1). The directions of odds were similar for ROA and knee ROA when using 30 ng/mL as the cutoff, though not reaching statistical significance possibly due to the small number of participants with 25(OH)D ≥ 30 ng/mL (*n* = 515 [267 men and 248 women]; data not shown). On the other hand, the odds of lumbar spine ROA, severe knee ROA (KL grade ≥ 3 vs 2), and presence of symptomatic OA (at the knee, lumbar spine, or hip; Supplementary figure) were not associated with vitamin D status.

**Table 2:** Crude and adjusted ORs (95% CI) of ROA by 25(OH)D status in men 50 years and older from KNHANES 2010–2013 (*n* = 3830) stratified by age or BMI.

25(OH)D (ng/mL)	Age						BMI					
	< 65 years			≥ 65 years			< 23			≥ 23		
	*n*	Crude OR (95% CI)	Adjusted OR (95% CI)	*n*	Crude OR (95% CI)	Adjusted OR (95% CI)	*n*	Crude OR (95% CI)	Adjusted OR (95% CI)	*n*	Crude OR (95% CI)	Adjusted OR (95% CI)
**ROA**												
< 12	200	0.83 (0.54–1.30)	0.99 (0.63–1.55)	159	0.55 (0.33–0.93)	0.61 (0.36–1.03)	166	0.78 (0.47–1.28)	0.85 (0.47–1.53)	193	0.74 (0.48–1.14)	0.79 (0.49–1.27)
12–20	1048	0.68 (0.53–0.86)	0.74 (0.57–0.96)	718	0.83 (0.61–1.13)	0.87 (0.63–1.20)	647	0.80 (0.59–1.09)	0.89 (0.63–1.25)	119	0.67 (0.53–0.83)	0.72 (0.56–0.93)
≥ 20	934	1	1	771	1	1	668	1	1	1037	1	1
*P* for trend		0.008	0.06		0.064	0.18		0.33	0.76		0.001	0.04
**LS ROA**												
< 12	200	1.18 (0.71–1.98)	0.77 (0.49–1.21)	159	0.65 (0.39–1.08)	0.71 (0.45–1.11)	166	0.73 (0.42–1.27)	0.76 (0.41–1.38)	193	1.10 (0.70–1.75)	1.14 (0.68–1.93)
12–20	1048	0.84 (0.63–1.13)	0.89 (0.64–1.24)	718	0.83 (0.62–1.11)	0.96 (0.69–1.35)	647	0.83 (0.61–1.140	0.85 (0.60–1.20)	119	0.80 (0.63–1.03)	0.85 (0.65–1.12)
≥ 20	934	1	1	771	1	1	668	1	1	1037	1	1
*P* for trend		0.29	0.52		0.19	0.27		0.38	0.53		0.15	0.33
**Knee ROA**												
< 12	200	0.60 (0.33–1.11)	0.73 (0.39–1.36)	159	0.63 (0.37–1.07)	0.74 (0.42–1.3)	166	0.76 (0.44–1.33)	0.90 (0.47–1.73)	193	0.60 (0.34–1.04)	0.67 (0.37–1.2)
12–20	1048	0.52 (0.37–0.72)	0.56 (0.39–0.8)	718	0.93 (0.39–1.26)	1.05 (0.76–1.44)	647	0.77 (0.53–1.13)	0.93 (0.61–1.41)	119	0.60 (0.46–0.77)	0.67 (0.5–0.9)
≥ 20	934	1	1	771	1	1	668	1	1	1037	1	1
*P* for trend		0.0004	0.006		0.22	0.48		0.37	0.92		0.0003	0.02

BMI was categorized as underweight/normal weight (< 23) and overweight/obese (≥ 23) according to the World Health Organization overweight and obesity criteria for Asians^49^. ROA indicates at least one incidence of ROA at the knee, lumbar spine, or hip confirmed by X-ray. Logistic regression was used for analyses. All models were adjusted for clustering and stratification. Adjusted models were adjusted for age, income, education level, longest job, physical activity, smoking status, alcohol consumption, BMI (when stratified by age), season of blood draw, and survey year. Weights were applied according to the guidelines of the KNHANES. BMI, body mass index; CI, confidence interval; KNHANES, Korea National Health and Nutrition Examination Survey; LS, lumbar spine; OR, odds ratio; ROA, radiologic osteoarthritis; 25(OH)D, 25-hydroxyvitamin D.

To assess whether the positive association between ROA and vitamin D status is owing to increased health-promoting behaviors of patients with ROA as an effort to attenuate the disease, we determined the relationship between ROA and self-reported OA. Among those with ROA, 89.5% of men and 62.2% of women were unaware of its presence (Table 3). **S**elf-reporting of OA had low sensitivity (27.4%). These results were similar when participants for whom 25(OH)D data were absent were included (data not shown). Knee pain sensitivity and specificity were 37.1% and 86.3% for knee ROA, respectively (Supplementary Table 2).

**Table 3:** Agreement between radiographic and self-reported OA in adults 50 years and older in KNHANES 2010–2013.

Method of assessment		Total (*n* = 8575)	Men (*n* = 3830)	Women (*n* = 4745)
Radiographic OA^a^	Self-reported OA	*n* (%)	*n* (%)	*n* (%)
Negative	Negative	3809 (48.6)	2055 (57.9)	1754 (40.3)
	Positive	319 (3.7)	83 (2.1)	236 (5.2)
Positive	Negative	3228 (34.5)	1513 (35.5)	1715 (33.6)
	Positive	1219 (13.2)	179 (4.5)	1040 (20.9)
Sensitivity (%)		27.4	10.5	37.8
Specificity (%)		92.3	96.1	88.1
Positive predictive value (%)		79.3	68.3	81.5
Negative predictive value (%)		54.1	57.6	50.6

Radiographic OA is the gold standard to diagnose OA. Data are shown as unweighted *n* (weighted percentage). Sample weights were applied according the directions of the KNHANES. KNHANES, Korea National Health and Nutrition Examination Survey; OA, osteoarthritis.

^a^Joints assessed for radiographic OA include the knee, lumbar spine, and hip.

## Discussion

We assessed the association of OA with vitamin D status at relatively low cutoffs using a nationally representative database of Koreans. Men with 25(OH)D < 20 ng/mL had a lower odds of knee ROA than those with a higher status. Serum 25(OH)D was not associated with the severity of knee ROA or presence of lumbar spine ROA or symptomatic OA. Sensitivity for ROA was 27%.

An unexpected positive association between vitamin D status and the odds of ROA was detected in Korean men. It is very unlikely that ROA increased vitamin D status. The low proportion (10%) of men aware of their ROA and the lack of association between self-reported OA and vitamin D status indicate that knowledge of one’s ROA most likely did not affect vitamin D status or the higher odds of ROA with higher vitamin D status. Therefore the results of this study are unlikely to be due to reverse causation. Although it is unlikely that men with ROA intentionally used more vitamin D supplements, the Korea National Health and Nutrition Examination Survey (KNHANES) database does not provide information on vitamin D supplement intake to confirm this assumption. On the other hand, further evaluation is required to test if vitamin D metabolites cause OA. One in vitro study indicates the active vitamin D metabolite, 1,25-dihydroxyvitamin D_3_ (1,25(OH)_2_D_3_), activates matrix metallopeptidase 13 expression, which plays a major role in cartilage degradation^19^, while another reported that 1,25(OH)_2_D_3_ suppresses OA by increasing the expression of Sox 9, a marker of chondrocyte differentiation^20^. In addition, the joint may be affected by vitamin D metabolites of non-canonical pathways^21,22^. The relationship between serum 25(OH)D and vitamin D metabolites, such as 20-hydroxyvitamin D, 22-hydroxyvitamin D, and their derivatives, require more investigation. These analogues have been reported to be non- or low- calcemic and can activate lumisterol^23,24^; however further research on their potential role in the joint is needed. Previous prospective observational studies resulted in a null or reverse association between vitamin D status and the risk of OA incidence in Whites^3–6^. The different results between previous studies and the current study may be due to race, which has been reported to be associated with the prevalence of OA^25^. This may be especially true as Koreans have a high occurrence of knee ROA^26^ but relatively low vitamin D status^27^ compared to other Western countries. Genetic polymorphisms associated with OA differ between Whites and Japanese adults^12^. In addition, polymorphisms of the VDR were not associated with risk of OA in Europeans whereas the VDR Apa1 gene polymorphism was associated with OA in Asians, indicating that Asians may be more sensitive to vitamin D status and metabolism than Whites regarding OA^13^. No studies have compared the relationship between vitamin D status and OA initiation among races. Whether high vitamin D status causes ROA in Korean men must be further investigated in prospective studies.

Regarding progression of ROA, the null association between vitamin D status and ROA severity are remarkably consistent with the lack of effect of vitamin D supplementation in OA patients in previous RCTs performed in Whites^7–9^ despite the cross-sectional design of this study. These results indicate that racial differences may not be present in the association between vitamin D status and progression of OA. The baseline 25(OH)D levels of the participants of previous RCTs were relatively high, but our study additionally shows that a lower vitamin D status is unlikely to affect ROA progression. Previous RCTs also showed no effect of vitamin D supplementation on knee pain^7–9,16^. Although a slight improvement in clinical symptoms was observed in adults with 25(OH)D < 20 ng/mL in one study^15^, whether this change is clinically meaningful is debatable. Our study findings indicate that increasing one’s 25(OH)D to > 20 ng/mL may not affect knee pain. Still, due to the possible racial differences in vitamin D metabolism and OA, future prospective studies in Koreans are required to fully understand the effect of vitamin D on OA progression and clinical symptoms.

The distinct differences (OA prevalence, awareness, presence of pain, and association with vitamin D status) between the sexes suggest differences in the causes and phenotypes of OA. The lack of an association between vitamin D and ROA in women versus the presence in men indicates that hormonal differences or changes may override the association between vitamin D and ROA. Many studies, including ours, reported a higher prevalence of OA in women than in men, although these may differ according to the joint examined^28^. Menopausal status and estrogen are also known to affect OA initiation and progression^29^. Previous studies investigating the relationship between vitamin D and ROA did not stratify the population by sex^4–9,15,16^, and most animal research on OA was performed in male animals. Therefore, the present study indicates a need for sex-specific research into the effect of vitamin D status on OA incidence and progression.

Although the discordance between ROA and self-reported OA has been anecdotally reported in Koreans, this is the first study to document the rate of discordance in Koreans. Most self-reports of OA may be based on joint pain and consequent self-diagnosis of OA and not professional diagnosis, possibly due to high medical cost, low accessibility to medical facilities, high availability of remedies for pain management, etc. On the other hand, those without joint pain may not be motivated to seek medical attention for OA, resulting in the low sensitivity as observed in the present study (Table 3). As pain sensitivity and management differ according to race and ethnicity, the inconsistency between self-reported OA and ROA may vary among different ethnicities and races^30^. However, the inconsistency between ROA and self-reported OA in Koreans in this study are similar to that reported in a study conducted in the UK^31^. The surprisingly similar discrepancy between self-reported OA and ROA among adults indicates that the inaccuracy of self-reported OA is preserved to similar degrees across cultures regardless of prevalence of ROA.

Our study has several limitations. First, a causal relationship cannot be confirmed due to its cross-sectional design. However, by assessing the agreement between ROA and self-reported OA, we were able to weaken the concern of possible reverse causation. As vitamin D intake data are not available and information on sunlight exposure behaviors are limited in the KNHANES database, we are unable to confirm whether adults with ROA consumed more vitamin D or participated in more outdoor activities than those without. In addition, the cross-sectional design may include additional potential confounders. For instance, meat consumption has been associated with OA^32^, possibly due to cholesterol intake and metabolism^33^. However, meat also contains vitamin D and 25(OH)D^34,35^ which may explain the higher 25(OH)D in meat and fish eaters compared to vegetarians and vegans^36^. The positive relationship between vitamin D and OA in Korean men may be due to their higher consumption of meat compared to women^37^. However we were unable to assess dietary intake in this study. Second, the target population of the KNHANES is non-institutionalized civilians, which excludes older adults in nursing homes where many OA patients may reside. However, adults dwelling in health care facilities are more likely to have serious comorbidities that may obscure the relationship between vitamin D and OA. Third, previous reports indicate that vitamin K may interact with vitamin D and OA^38^, but the current KNHANES database does not include vitamin K intake or serum vitamin K metabolite data. The mean vitamin K intake of Korean adults 50 years and older is well over the Adequate Intakes for Koreans^39^; thus, vitamin K may not have a strong effect in our population. Still, the interaction between vitamin D and vitamin K on OA in Koreans requires examination. Fourth, although radiography is the basic diagnostic tool for OA, it may neglect small osteophytes or cartilage injuries. However, the KNHANES data did not include OA evaluation using advanced imaging techniques such as computed tomography or magnetic resonance imaging due to high cost and transportation difficulties. Nevertheless KNHANES determined OA using both weight-bearing anteroposterior and mediolateral images of the joints to minimize error in the diagnosis of ROA. Despite these limitations, our study is the first to comprehensively analyze the relationship between vitamin D status and ROA according to sex utilizing a nationally representative database of Asians. Our data highlight the need of prospective longitudinal studies in Asians on vitamin D status and OA.

In older Korean adults, the prevalence of ROA was 47%. Lower vitamin D status (< 20 ng/mL) was associated with a lower odds of ROA in Korean men, but not women. Knee ROA severity or presence of pain were not associated with vitamin D status. Agreement of self-reported OA and ROA were low in men with ROA, indicating that knowledge of OA may not have affected the results. Vitamin D may be involved in OA initiation in but not progression in Korean men, but prospective studies are required to confirm causality.

## Methods

### Study population

**Figure 2:**
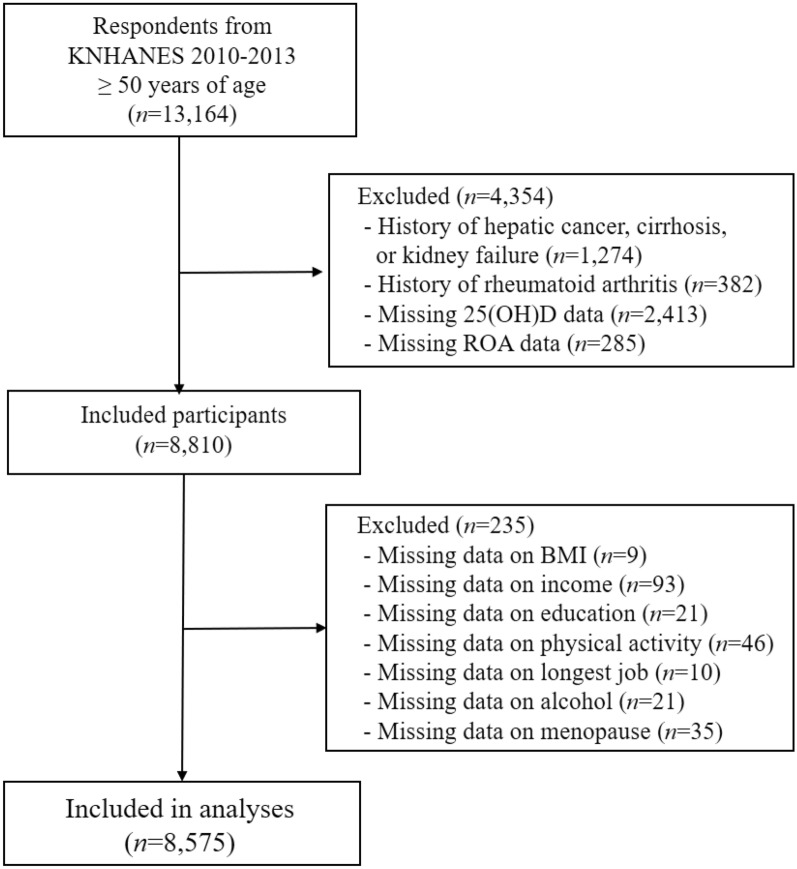
Flow diagram of participants included in the analyses. BMI, body mass index; KNHANES, Korea National Health and Nutrition Examination Survey; ROA, radiologic osteoarthritis; 25(OH)D, 25-hydroxyvitamin D.

The KNHANES provides a cross-sectional representation of the health status and nutrition-related behaviors of the non-institutionalized population of Korea. Years 2010–2013 of the KNHANES database (accessed March 27, 2020) was used^40^. Korean adults aged 50 years and older were selected. The criteria and number of subjects selected to assess the likelihood of OA are described in Fig. 2. Subjects with hepatic cancer, cirrhosis, or kidney failure were removed as vitamin D metabolism may be affected by these diseases. Participants with a history of rheumatoid arthritis (RA) were also excluded to prevent confusion of joint pain due to RA. Participants excluded from the analyses had higher proportions of females, smokers, non-drinkers, postmenopausal women, and those with lower income, lower education status, and less physical activity than adults included in the analyses. Mean 25(OH)D was slightly lower in excluded adults than included adults (17.9 ± 0.3 vs 18.9 ± 0.2 ng/mL, *P* = 0.0012). Data analyzed in this study are publicly available and anonymized before access. The Korea Disease Control and Prevention Agency (KCDA) Institutional Review Board (IRB) approved the protocol for all KNHANES content^40^ while the protocol of this study was approved by the IRB of Chonnam National University (IRB number: 1040198-170314-HR-007-01). All methods were performed in accordance with the Declaration of Helsinki and relevant regulations. Informed consent was obtained from all participants by the KCDA.

### Determination of OA

ROA: X-ray images (DigiRAD-PG; Sitec Medical Co., Kimpo, Korea) of the lumbar spine (LS; anteroposterior and lateral), knee (bilateral anteroposterior, lateral, and weight-bearing anteroposterior), and hip joints (anteroposterior pelvis and upper femoral, anteroposterior, and oblique hip joint) were examined. The radiographs were independently assessed by two trained radiologists^41^. Lumbar spine radiographs were assessed by the scale developed by The Korean Academy of Family Medicine using the scale by Yoshimura et al.^42^ (range, 0–2) based on Kellgren–Lawrence (KL) classification^43^. Radiographs of the knee were assessed for KL grade (range, 0–4) and hip joints were graded according to American College of Rheumatology guidelines (range, 0–3)^44,45^. Radiographic OA was defined as grade ≥ 2. If ROA was present in at least one site among the LS, knee, or hip, the participant was categorized as having ROA. When ROA was not present at any of the study sites, the participant was categorized as not having ROA. In the knee ROA severity analysis, participants were grouped as KL grade 2 or ≥ 3.

Symptomatic OA: Participants with ROA and joint pain at the corresponding site for more than 30 days in the past 3 months regardless of medication use or self-report of OA were identified as having symptomatic OA.

Knee pain: Among participants with self-reported OA (*n* = 1677), those who reported having knee pain for more than 30 days in the past 3 months regardless of medication use were categorized as having knee pain. Knee pain severity was evaluated using an 11-point numerical rating scale (0, no pain; 10, severe pain). Knee pain level was analyzed as a continuous value or categorized into two groups (mild, 1–5; severe, 6–10) for the severity analysis.

Self-reported OA: If a participant answered “yes” to the question “Do you currently have osteoarthritis?” during the health interview, the participant was identified as having self-reported OA.

### Measurement of serum 25(OH)D

Fasting blood samples (≥ 8 h overnight fast) were collected to measure serum 25(OH)D by ^125^I-labelled radioimmunoassay (Diasorin, Stillwater, MN, USA). Serum 25(OH)D status was categorized into three groups (< 12 ng/mL, 12 to < 20 ng/mL, and ≥ 20 ng/mL) or two groups (< 20 and ≥ 20 ng/mL) according to sample size and based on the current vitamin D recommendations for Koreans^46^ and North Americans^47^.

### Other characteristics

Height and weight were measured on a stadiometer (Seca 225; Seca, Hamburg, Germany) and weight scale (GL-6000–20; G-tech, Seoul, Korea), respectively, to calculate body mass index (BMI). Information on age, household income, education level, and longest job were collected during the health interview. Household equivalent median income (HEMI) was calculated using the following equation:

$$\mathrm{HEMI }(\mathrm{\%})=\frac{\mathrm{total \; household \; income}}{\sqrt{\mathrm{family \; members }}\times \mathrm{equivalent \; median \;disposable \; income}}\times 100$$

The equivalent median disposable incomes for each year was determined from the Korean Statistical Information Service (KOSIS) website^48^. Total household income was bottom-coded at 170,000 KRW and top-coded at 15 million KRW. Educational level was classified into three groups (elementary school graduate or lower, middle school graduate, and high school graduate or above) or two groups (elementary school graduate and lower, and middle school graduate and above) to ensure a sufficient number of subjects. Each participant’s longest job was documented to determine long-term physical activity level. Occupation was classified by the Korean Standard Classification of Occupations and categorized into two groups: clerical workers (e.g., sales, service, clerical work, research, medicine, law, education, administration) and manual workers (e.g., agriculture, forestry, fishery, engineering, assembling, technical work, military work, manual labor) as previously reported by Seo et al.^49^ or three groups (clerical workers, manual workers, and other [unemployed and housewives]).

Health behavior data, including physical activity, smoking status, alcohol consumption, and menopausal status, were collected through a self-administered questionnaire. Physical activity intensity was classified according to the KNHANES categorization scheme and the World Health Organization physical activity guidelines^50^. Duration of physical activity was calculated as the minutes of moderate activity summed with two times the duration of vigorous activity performed during the past week. Smoking status was classified as current or previous smokers and never smokers. Frequency of alcohol consumption was classified into two groups: less than once per month versus once or more per month during the previous year. Menopausal status was categorized as premenopausal or postmenopausal (including both natural and surgical menopause) according to information from the health behavior survey and self-reported data from the bone mineral density examination survey (KNHANES 2009–2011). Season of blood draw was categorized into two groups (June to November and May to December) based on mean monthly serum 25(OH)D concentrations in Koreans^51^.

### Statistical analyses

All analyses were performed in the total population and then stratified by sex. Adults were additionally stratified by age (< or ≥ 65 years of age) or BMI within each sex for ROA. BMI was stratified as < 23 (underweight or normal weight) or ≥ 23 (overweight/obese), according to the World Health Organization overweight and obesity criteria for Asians^52^. The number of participants with severe ROA or symptomatic OA was inadequate to additionally stratify within each sex. Differences in participant characteristics were investigated according to OA status using Rao-Scott *χ*^2^ analysis or independent *t*-test. Natural log transformation was applied for non-normally distributed continuous variables. Standard error is reported as this is a nationally representative sample. To assess the odds of OA prevalence, progression, and clinical symptoms by vitamin D status, odds ratio (ORs) were determined by logistic regression analysis and expressed with 95% confidence intervals (CIs). All logistic regression models were adjusted for sex (total population), age, BMI, HEMI, education level, physical activity, longest job, smoking status, alcohol consumption, season of blood draw, survey year, and menopausal status (women). The number of hip OA patients was too small to analyze independently. The analyses were performed using PROC SURVEYMEANS, PROC SURVEYREG and PROC SURVEYLOGISTIC in SAS 9.4 (SAS Institute Inc., Cary, NC, USA) considering strata, cluster, and weight provided in the KNHANES database and calculated according to the directions of the KNHANES^40^. Sensitivity and specificity were assessed following the methods of Parsons et al.^31^. Sensitivity indicates the proportion or those with ROA that also self-report to have OA. Specificity is the proportion of those without ROA that report oneself to be absent of OA. Null hypotheses were rejected at values of *P* < 0.05.

## Supplementary Information

Supplementary Information.

## Data Availability

Datasets analyzed during the current study are publically available at the Korea National Health and Nutrition Examination Survey website (https://knhanes.kdca.go.kr/knhanes/sub03/sub03_02_05.do).

## References

[1] OARSI. Is Osteoarthritis a Serious Disease? (Osteoarthritis Research Society International, 2020) https://oarsi.org/research/infographic-oa-serious-disease (Accessed 1 Dec 2021).

[2] Sellam J, Berenbaum F (2013). Is osteoarthritis a metabolic disease?. Joint Bone Spine.

[3] Ding C, Cicuttini F, Parameswaran V, Burgess J, Quinn S, Jones G (2009). Serum levels of vitamin D, sunlight exposure, and knee cartilage loss in older adults: The Tasmanian older adult cohort study. Arthritis Rheum..

[4] Felson DT, Niu J, Clancy M, Aliabadi P, Sack B, Guermazi A, Hunter DJ, Amin S, Rogers G, Booth SL (2007). Low levels of vitamin D and worsening of knee osteoarthritis: Results of two longitudinal studies. Arthritis Rheum..

[5] Konstari S, Paananen M, Heliövaara M, Knekt P, Marniemi J, Impivaara O, Arokoski J, Karppinen J (2012). Association of 25-hydroxyvitamin D with the incidence of knee and hip osteoarthritis: A 22-year follow-up study. Scand. J. Rheumatol..

[6] McAlindon TE, Felson DT, Zhang Y, Hannan MT, Aliabadi P, Weissman B, Rush D, Wilson PW, Jacques P (1996). Relation of dietary intake and serum levels of vitamin D to progression of osteoarthritis of the knee among participants in the Framingham Study. Ann. Intern. Med..

[7] Arden NK, Cro S, Sheard S, Doré CJ, Bara A, Tebbs SA, Hunter DJ, James S, Cooper C, O'Neill TW (2016). The effect of vitamin D supplementation on knee osteoarthritis, the VIDEO study: A randomised controlled trial. Osteoarthr. Cartil..

[8] Jin X, Jones G, Cicuttini F, Wluka A, Zhu Z, Han W, Antony B, Wang X, Winzenberg T, Blizzard L (2016). Effect of vitamin D supplementation on tibial cartilage volume and knee pain among patients with symptomatic knee osteoarthritis: A randomized clinical trial. J. Am. Med. Assoc..

[9] McAlindon T, LaValley M, Schneider E, Nuite M, Lee JY, Price LL, Lo G, Dawson-Hughes B (2013). Effect of vitamin D supplementation on progression of knee pain and cartilage volume loss in patients with symptomatic osteoarthritis: A randomized controlled trial. J. Am. Med. Assoc..

[10] Nevitt MC, Xu L, Zhang Y, Lui LY, Yu W, Lane NE, Qin M, Hochberg MC, Cummings SR, Felson DT (2002). Very low prevalence of hip osteoarthritis among Chinese elderly in Beijing, China, compared with whites in the United States: The Beijing osteoarthritis study. Arthritis Rheum..

[11] Zhang Y, Xu L, Nevitt MC, Aliabadi P, Yu W, Qin M, Lui LY, Felson DT (2001). Comparison of the prevalence of knee osteoarthritis between the elderly Chinese population in Beijing and whites in the United States: The Beijing Osteoarthritis Study. Arthritis Rheum..

[12] Valdes AM, Loughlin J, Oene MV, Chapman K, Surdulescu GL, Doherty M, Spector TD (2007). Sex and ethnic differences in the association of ASPN, CALM1, COL2A1, COMP, and FRZB with genetic susceptibility to osteoarthritis of the knee. Arthritis Rheum..

[13] Zhu Z-H, Jin X-Z, Zhang W, Chen M, Ye D-Q, Zhai Y, Dong F-L, Shen C-L, Ding C (2014). Associations between vitamin D receptor gene polymorphisms and osteoarthritis: An updated meta-analysis. Rheumatology.

[14] Glover T, Goodin B, Horgas A, Kindler L, King C, Sibille K, Peloquin C, Riley J, Staud R, Bradley L, Vitamin D (2012). race, and experimental pain sensitivity in older adults with knee osteoarthritis. Arthritis Rheum..

[15] Sanghi D, Mishra A, Sharma AC, Singh A, Natu S, Agarwal S, Srivastava RN (2013). Does vitamin D improve osteoarthritis of the knee: A randomized controlled pilot trial. Clin. Orthop. Relat. Res..

[16] Zheng S, Jin X, Cicuttini F, Wang X, Zhu Z, Wluka A, Han W, Winzenberg T, Antony B, Aitken D (2017). Maintaining vitamin D sufficiency is associated with improved structural and symptomatic outcomes in knee osteoarthritis. Am. J. Med..

[17] Um J, Zaidi A, Choi SJ (2019). Active Ageing Index in Korea–comparison with China and EU countries. Asian Soc. Work Policy Rev..

[18] Yoo K, Cho J, Ly S (2016). Vitamin D intake and serum 25-hydroxyvitamin D levels in Korean adults: Analysis of the 2009 Korea National Health and Nutrition Examination Survey (KNHANES IV-3) using a newly established vitamin D database. Nutrients.

[19] Chen D, Li Y, Dai X, Zhou X, Tian W, Zhou Y, Zou X, Zhang C (2013). 1, 25-Dihydroxyvitamin D3 activates MMP13 gene expression in chondrocytes through p38 MARK pathway. Int. J. Biol. Sci..

[20] Hdud IM, Loughna PT (2014). Influence of 1α, 25-dihydroxyvitamin D 3 [1,25(OH)2D3] on the expression of Sox 9 and the transient receptor potential vanilloid 5/6 ion channels in equine articular chondrocytes. J. Anim. Sci. Technol..

[21] Slominski AT, Kim TK, Shehabi HZ, Semak I, Tang EK, Nguyen MN, Benson HA, Korik E, Janjetovic Z, Chen J (2012). In vivo evidence for a novel pathway of vitamin D3 metabolism initiated by P450scc and modified by CYP27B1. FASEB J..

[22] Slominski AT, Kim T-K, Li W, Postlethwaite A, Tieu EW, Tang EK, Tuckey RC (2015). Detection of novel CYP11A1-derived secosteroids in the human epidermis and serum and pig adrenal gland. Sci. Rep..

[23] Slominski AT, Kim T-K, Hobrath JV, Janjetovic Z, Oak ASW, Postlethwaite A, Lin Z, Li W, Takeda Y, Jetten AM, Tuckey RC (2017). Characterization of a new pathway that activates lumisterol in vivo to biologically active hydroxylumisterols. Sci. Rep..

[24] Slominski AT, Li W, Kim T-K, Semak I, Wang J, Zjawiony JK, Tuckey RC (2015). Novel activities of CYP11A1 and their potential physiological significance. J. Steroid Biochem. Mol. Biol..

[25] Spector TD, MacGregor AJ (2004). Risk factors for osteoarthritis: Genetics. Osteoarthr. Cartil..

[26] Cui A, Li H, Wang D, Zhong J, Chen Y, Lu H (2020). Global, regional prevalence, incidence and risk factors of knee osteoarthritis in population-based studies. EClinicalMedicine.

[27] Van Schoor N, Lips P (2017). Global overview of vitamin D status. Endocrinol. Metab. Clin..

[28] Srikanth VK, Fryer JL, Zhai G, Winzenberg TM, Hosmer D, Jones G (2005). A meta-analysis of sex differences prevalence, incidence and severity of osteoarthritis. Osteoarthr. Cartil..

[29] Sniekers Y, Weinans H, Bierma-Zeinstra S, Van Leeuwen J, Van Osch G (2008). Animal models for osteoarthritis: The effect of ovariectomy and estrogen treatment—A systematic approach. Osteoarthr. Cartil..

[30] Campbell CM, Edwards RR (2012). Ethnic differences in pain and pain management. Pain Manag..

[31] Parsons C, Clynes M, Syddall H, Jagannath D, Litwic A, van der Pas S, Cooper C, Dennison EM, Edwards MH, Group ER (2015). How well do radiographic, clinical and self-reported diagnoses of knee osteoarthritis agree? Findings from the Hertfordshire cohort study. Springerplus.

[32] Hailu A, Knutsen S, Fraser G (2006). Associations between meat consumption and the prevalence of degenerative arthritis and soft tissue disorders in the adventist health study, California USA. J. Nutr. Health Aging.

[33] Choi W-S, Lee G, Song W-H, Koh J-T, Yang J, Kwak J-S, Kim H-E, Kim SK, Son Y-O, Nam H (2019). The CH25H–CYP7B1–RORα axis of cholesterol metabolism regulates osteoarthritis. Nature.

[34] Dunlop E, James AP, Cunningham J, Strobel N, Lucas RM, Kiely M, Nowson CA, Rangan A, Adorno P, Atyeo P (2021). Vitamin D composition of Australian foods. Food Chem..

[35] Cashman KD, O'Sullivan SM, Galvin K, Ryan M (2020). Contribution of vitamin D2 and D3 and their respective 25-hydroxy metabolites to the total vitamin D content of beef and lamb. Curr. Dev. Nutr..

[36] Crowe FL, Steur M, Allen NE, Appleby PN, Travis RC, Key TJ (2011). Plasma concentrations of 25-hydroxyvitamin D in meat eaters, fish eaters, vegetarians and vegans: Results from the EPIC–Oxford study. Public Health Nutr..

[37] Kim KW, Park HA, Cho YG, Bong AR (2021). Protein intake by Korean adults through meals. Korean J. Health Promot..

[38] Shea MK, Loeser RF, McAlindon TE, Houston DK, Kritchevsky SB, Booth SL (2018). Association of Vitamin K status combined with Vitamin D status and lower-extremity function: A prospective analysis of two knee osteoarthritis cohorts. Arthritis Care Res..

[39] Kim E-S, Kim M-S, Na W-R, Sohn C-M (2013). Estimation of vitamin K intake in Koreans and determination of the primary vitamin K-containing food sources based on the fifth Korean National Health and Nutrition Examination Survey (2010–2011). Nurs. Res. Pract..

[40] The Korea National Health and Nutrition Examination Survey (KNHANES) [Internet]. Korea, Chungcheongbuk-do, Cheongju (28159): Korea Disease Control and Prevention Agency. https://knhanes.kdca.go.kr/knhanes/sub03/sub03_02_05.do (Accessed 29 Nov 2021).

[41] KCDC. Korea National Health and Nutrition Examination Survey VI, the first year (2013): Professional Surveyor Education and Quality Control for Osteoarthritis Examination. Korea, Chungcheongbuk-do, Cheongju (28159): Korea Centers for Disease Control and Prevention (2013).

[42] Yoshimura N, Muraki S, Oka H, Mabuchi A, En-Yo Y, Yoshida M, Saika A, Yoshida H, Suzuki T, Yamamoto S (2009). Prevalence of knee osteoarthritis, lumbar spondylosis, and osteoporosis in Japanese men and women: The research on osteoarthritis/osteoporosis against disability study. J. Bone Miner. Metab..

[43] Kellgren J, Lawrence J (1957). Radiological assessment of osteo-arthrosis. Ann. Rheum. Dis..

[44] Altman R, Asch E, Bloch D, Bole G, Borenstein D, Brandt K, Christy W, Cooke T, Greenwald R, Hochberg M (1986). Development of criteria for the classification and reporting of osteoarthritis: Classification of osteoarthritis of the knee. Arthritis Rheum..

[45] Altman R, Alarcon G, Appelrouth D, Bloch D, Borenstein D, Brandt K, Brown C, Cooke T, Daniel W, Feldman D (1991). The American College of Rheumatology criteria for the classification and reporting of osteoarthritis of the hip. Arthritis Rheum..

[46] Dietary reference intakes for Koreans. (Ministry of Health & Welfare, The Korean Nutrition Society, 2015).

[47] Dietary Reference Intakes for Calcium and Vitamin D. (The National Academies Press: IOM, Institute of Medicine); 2011. 21796828

[48] KOSIS. Distribution of Income Index. Statistics Korea; 2008–2014. http://kosis.kr/statHtml/statHtml.do?orgId=101&tblId=DT_1L6E001 (Accessed 15 May 2020).

[49] Seo MH, Kim MK, Park SE, Rhee EJ, Park CY, Lee W-Y, Baek KH, Song K, Kang MI, Oh KW (2013). The association between daily calcium intake and sarcopenia in older, non-obese Korean adults: The fourth Korea National Health and Nutrition Examination Survey (KNHANES IV) 2009. Endocr. J..

[50] WHO. Global recommendations on physical activity for health. Sydney: Health Communications Australia (World Health Organization, 2010). 26180873

[51] Choi HS, Oh HJ, Choi H, Choi WH, Kim JG, Kim KM, Kim KJ, Rhee Y, Lim S-K (2011). Vitamin D insufficiency in Korea—a greater threat to younger generation: The Korea National Health and Nutrition Examination Survey (KNHANES) 2008. J. Clin. Endocrinol. Metab..

[52] WHO. The Asia-Pacific perspective: redefining obesity and its treatment (2000).

