# Supplementary material for: Older Korean men with inadequate vitamin D status have lower odds of radiologic osteoarthritis
Source: Sci Rep. 2022 Jul 5;12:11372. doi: 10.1038/s41598-022-15025-9 (PMC9256662; doi:10.1038/s41598-022-15025-9)
Supplement: Supplementary file 1 — Supplementary Information. [file 41598_2022_15025_MOESM1_ESM.docx]

|  | Age | | | | | | |  | BMI | | | | | |
| --- | --- | --- | --- | --- | --- | --- | --- | --- | --- | --- | --- | --- | --- | --- |
|  | < 65 years | | | < 65 years | | | |  | < 23 | | | ≥ 23 | | |
| 25(OH)D (ng/mL) | *n* | Crude OR  (95% CI) | Adjusted OR  (95% CI) | | *n* | Crude OR  (95% CI) | Adjusted OR  (95% CI) |  | *n* | Crude OR  (95% CI) | Adjusted OR  (95% CI) | *n* | Crude OR  (95% CI) | Adjusted OR  (95% CI) |
| ROA^b^ |  |  |  | |  |  |  |  |  |  |  |  |  |  |
| <12 | 446 | 0.77 | 0.78 | | 338 | 0.80 | 0.72 |  | 288 | 0.69 | 0.91 | 496 | 0.76 | 0.81 |
|  |  | (0.54-1.10) | (0.54-1.14) | |  | (0.46-1.40) | (0.4-1.3) |  |  | (0.42-1.05) | (0.54-1.53) |  | (0.53-1.10) | (0.51-1.27) |
| 12-20 | 1504 | 0.74 | 0.74 | | 909 | 1.17 | 0.99 |  | 848 | 0.75 | 1.04 | 1565 | 0.71 | 0.84 |
|  |  | (0.57-0.95) | (0.57-0.96) | |  | (0.76-1.82) | (0.64-1.55) |  |  | (0.54-1.04) | (0.71-1.51) |  | (0.55-0.92) | (0.63-1.13) |
| ≥20 | 834 | 1 | 1 | | 714 | 1 | 1 |  | 588 | 1 | 1 | 960 | 1 | 1 |
| *P* for trend | | 0.06 | 0.07 | |  | 0.30 | 0.41 |  |  | 0.13 | 0.87 |  | 0.03 | 0.49 |
| LS ROA |  |  |  | |  |  |  |  |  |  |  |  |  |  |
| <12 | 446 | 0.73 | 0.74 | | 338 | 0.78 | 0.75 |  | 288 | 0.61 | 0.64 | 496 | 0.79 | 0.82 |
|  |  | (0.48-1.11) | (0.48-1.14) | |  | (0.51-1.20) | (0.5-1.14) |  |  | (0.38-0.96) | (0.38-1.09) |  | (0.55-1.13) | (0.54-1.23) |
| 12-20 | 1504 | 0.86 | 0.87 | | 909 | 1.07 | 0.96 |  | 848 | 0.84 | 1.01 | 1565 | 0.83 | 0.91 |
|  |  | (0.62-1.20) | (0.63-1.2) | |  | (0.78-1.47) | (0.7-1.31) |  |  | (0.59-1.19) | (0.68-1.50) |  | (0.63-1.08) | (0.68-1.22) |
| ≥20 | 834 | 1 | 1 | | 714 | 1 | 1 |  | 588 | 1 | 1 | 960 | 1 | 1 |
| *P* for trend | | 0.34 | 0.38 | |  | 0.28 | 0.36 |  |  | 0.10 | 0.18 |  | 0.29 | 0.61 |
| Knee ROA | |  |  | |  |  |  |  |  |  |  |  |  |  |
| <12 | 446 | 0.76 | 0.76 | | 338 | 0.85 | 0.76 |  | 288 | 0.65 | 0.84 | 496 | 0.79 | 0.84 |
|  |  | (0.51-1.14) | (0.5-1.16) | |  | (0.54-1.34) | (0.47-1.22) |  |  | (0.40-1.07) | (0.49-1.42) |  | (0.56-1.13) | (0.55-1.28) |
| 12-20 | 1504 | 0.74 | 0.75 | | 909 | 1.13 | 0.99 |  | 848 | 0.64 | 0.84 | 1565 | 0.82 | 0.98 |
|  |  | (0.56-0.98) | (0.56-0.99) | |  | (0.82-1.57) | (0.71-1.38) |  |  | (0.45-0.91) | (0.57-1.23) |  | (0.64-1.05) | (0.75-1.30) |
| ≥20 | 834 | 1 | 1 | | 714 | 1 | 1 |  | 588 | 1 | 1 | 960 | 1 | 1 |
| *P* for trend | | 0.09 | 0.12 | |  | 0.38 | 0.43 |  |  | 0.04 | 0.63 |  | 0.23 | 0.67 |

Supplementary table 1. Crude and adjusted ORs (95% CI) of ROA by 25(OH)D status in women 50 years and older from KNHANES 2010–2013 (*n* = 4745) stratified by age or BMI ^a)^

^a)^ BMI was categorized as underweight/normal weight (< 23) and overweight/obese (≥ 23) according to the World Health Organization overweight and obesity criteria for Asians [49]. ROA indicates at least one incidence of ROA at the knee, lumbar spine, or hip confirmed by X-ray. Logistic regression was used for analyses. All models were adjusted for clustering and stratification. Adjusted models were adjusted for age, income, education level, longest job, physical activity, smoking status, alcohol consumption, BMI (when stratified by age), menopausal status, season of blood draw, and survey year. Weights were applied according to the guidelines of the KNHANES. BMI, body mass index; CI, confidence interval; KNHANES, Korea National Health and Nutrition Examination Survey; LS, lumbar spine; OR, odds ratio; ROA, radiologic osteoarthritis; 25(OH)D, 25-hydroxyvitamin D

Supplementary table 2. Agreement between presence of knee pain and knee ROA in adults 50 years and older in KNHANES 2010–2013. ^a)^

| Method of assessment | | Total (*n* = 11583) | Men (*n* = 4923) | Women (*n* = 6660) |
| --- | --- | --- | --- | --- |
| Radiographic  knee OA | Self-reported  knee pain ^b)^ | *n* (%) | *n* (%) | *n* (%) |
| Negative | Negative | 6260 (57.1) | 3277 (69.5) | 2983 (46.6) |
|  | Positive | 994 (8.5) | 317 (6.6) | 677 (10) |
| Positive | Negative | 2723 (22) | 1033 (18.9) | 1690 (24.6) |
|  | Positive | 1606 (12.4) | 296 (5) | 1310 (18.7) |
| Sensitivity (%) | | 37.1 | 22.3 | 43.7 |
| Specificity (%) | | 86.3 | 91.2 | 81.5 |
| Positive predictive value (%) | | 61.8 | 48.3 | 65.9 |
| Negative predictive value (%) | | 69.7 | 76.0 | 63.8 |

^a)^ Radiographic OA is the gold standard to diagnose OA. Data are shown as unweighted *n* (weighted percentage). Sample weights were applied according the directions of the KNHANES. KNHANES, Korea National Health and Nutrition Examination Survey; OA, osteoarthritis

^b)^ Presence of knee pain is defined as reporting to have knee pain for more than 30 days in the past three months, regardless of medication use.


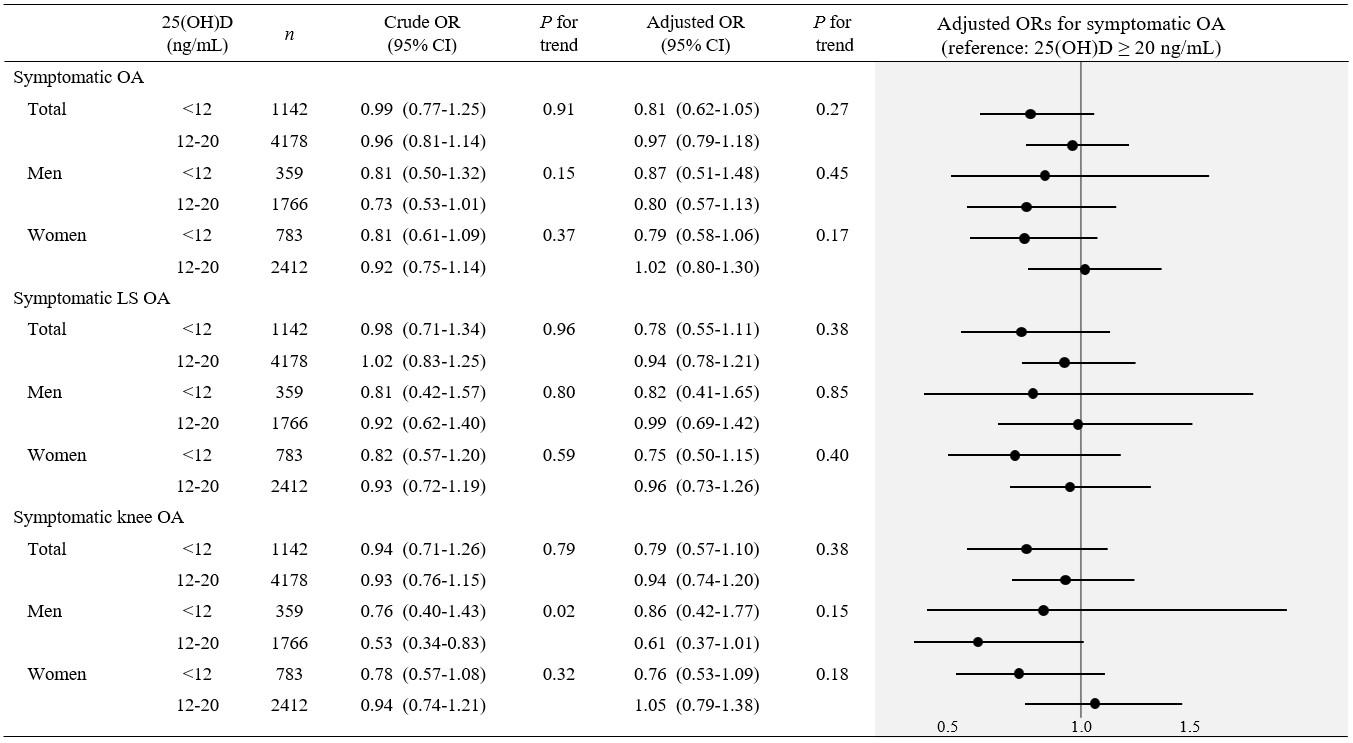


**Supplementary figure. Crude and adjusted ORs (95% CI) of symptomatic OA by 25(OH)D status in adults 50 years and older from KNHANES 2010–2013** Symptomatic OA indicates at least one incidence of radiologic OA at the knee, lumbar spine, or hip accompanied by pain at the corresponding joint. Sample size for the reference groups (25(OH)D ≥ 20 ng/mL) of symptomatic OA, symptomatic LS OA, and symptomatic knee OA for total, men, and women was 3253, 1705, and 1548, respectively. All models were adjusted for clustering and stratification. Adjusted models were adjusted for sex (total population), age, income, education level, longest job, physical activity, smoking status, alcohol consumption, body mass index, season of blood draw, survey year, and menopausal status (women). Weights were applied according to the guidelines of the KNHANES. CI, confidence interval; KNHANES, Korea National Health and Nutrition Examination Survey; LS, lumbar spine; OR, odds ratio; OA, osteoarthritis; 25(OH)D, 25-hydroxyvitamin D
